# Outcomes and Patient Satisfaction Following Micro-endoscopic Laminotomy for Lumbar Spinal Stenosis: A Comprehensive Analysis of Multiple Lumbar Spine Scoring Systems

**DOI:** 10.7759/cureus.97630

**Published:** 2025-11-24

**Authors:** Kohei Tsukahara, Teiyu Izumi, Shohei Yamada, Michinaga Yamashita, Keigo Shirasaki, Kimitaka Nakamura, Ryuta Imamura, Takahiro Hamada, Akihiko Inokuchi, Takeshi Arizono

**Affiliations:** 1 Orthopaedic Surgery, Kyushu Central Hospital of the Mutual Aid Association of Public School Teachers, Fukuoka, JPN

**Keywords:** japanese orthopaedic association score, lumbar spinal stenosis, micro-endoscopic laminotomy, oswestry disability index, patient satisfaction, roland-morris disability questionnaire, spine scoring system, spine surgery, surgical outcome, visual analogue scale

## Abstract

Aim: The aim of this study is to examine how patient-reported satisfaction at one year after micro-endoscopic laminotomy (MEL) for lumbar spinal stenosis (LSS) aligns with conventional outcome measures and to assess whether these instruments adequately reflect patient perspectives.

Materials and methods: We conducted a single-center retrospective cohort study of adults undergoing MEL for symptomatic LSS. Patients completed preoperative and one-year postoperative assessments, including visual analogue scales for pain and numbness, the Roland-Morris Disability Questionnaire (RMDQ), the Oswestry Disability Index (ODI), selected Japanese Orthopaedic Association (JOA) items, and the Japanese Orthopaedic Association Back Pain Evaluation Questionnaire (JOABPEQ). Patient satisfaction was captured by a recommendation-level item at one year. Pre-post changes and associations with satisfaction were analyzed using standard parametric or nonparametric tests as appropriate.

Results: One-year satisfaction was positively associated with postoperative functioning in social and psychological domains and with walking ability and pain-related impairment, and it was inversely associated with residual lower back pain and disability. Numbness showed a weaker association with satisfaction. Although conventional scales documented clinical improvement, they accounted for only part of the variance in satisfaction.

Conclusion: Conventional outcome scores capture important but incomplete aspects of recovery after MEL. Incorporating a brief, patient-reported satisfaction measure alongside established instruments can provide a more balanced appraisal of outcomes and support shared decision-making. Prospective studies are warranted to refine how satisfaction is integrated into routine evaluation.

## Introduction

Lumbar spinal stenosis (LSS) is a degenerative condition that causes lower back and leg pain, walking disabilities, decreased daily living functions, and locomotive syndrome. It is estimated that LSS affects approximately 3.65 million Japanese individuals between the ages of 40 and 79 years, with the prevalence rising to about 10% among those aged 70 to 79 years [1]. As the patient's age increases, so does the likelihood of complications, necessitating short-term bed rest and minimally invasive treatments. When the symptoms of LSS are severe and conservative treatments are ineffective, surgical intervention becomes the preferred option [2].

Since Foley and Smith introduced micro-endoscopic discectomy in 1997, the indications for endoscopic spinal surgeries have expanded significantly due to advancements in endoscopic equipment and surgical techniques [3]. Spinal endoscopic surgeries, including micro-endoscopic laminotomy (MEL) for LSS, have demonstrated favorable outcomes by achieving effective decompression with minimal invasiveness, which is crucial for older adult patients who are at higher risk of complications from traditional open surgeries [4].

The outcomes of surgery for LSS are commonly assessed using lumbar spine scoring systems. The Oswestry Disability Index (ODI) and Roland-Morris Disability Questionnaire (RMDQ) are widely used internationally, while the Japanese Orthopaedic Association (JOA) score is prevalently used in Japan and has shown a 60% improvement rate in postoperative outcomes [4,5,6,7]. However, the JOA score, while comprehensive, is not patient-centered. To address this, the JOA developed the Back Pain Evaluation Questionnaire (JOABPEQ) in 2009 to evaluate patient-specific quality of life measures for lumbar diseases [8].

Despite its validated reliability and validity, the JOABPEQ and other existing scoring systems may not adequately address the primary goal of surgery: patient satisfaction. This hypothesis is based on preliminary observations and reports indicating that patients’ subjective experiences and satisfaction may not be fully captured by these objective scoring systems. For example, while scores may indicate clinical improvement, patients often report dissatisfaction due to residual symptoms such as numbness or limitations in daily activities [9]. Previous studies have indicated a correlation between pain and treatment satisfaction and between psychosocial factors and patient satisfaction [4].

Patient satisfaction after LSS surgery has been reported in various studies, with satisfaction rates ranging from 60% to 80% depending on the specific criteria and methods used for assessment [10]. However, the variability in these rates underscores the need for standardized, reliable tools that accurately reflect patient satisfaction in a consistent manner [11].

The present study aimed to investigate the correlations between evaluations using existing lumbar spine scoring systems and patient-reported satisfaction after LSS surgery to determine whether these scores align with patient perspectives.

## Materials and methods

Study design and setting

This was a single-center retrospective cohort study of adults who underwent MEL for symptomatic LSS. Surgeries were performed between June 2020 and March 2021. Preoperative and one-year postoperative assessments were obtained as part of routine care and analyzed retrospectively.

Participants

Eligible patients were adults with clinical and radiographic LSS who underwent MEL. Exclusion criteria included patients with missing one-year postoperative assessments, those with recurrence of symptoms requiring re-operation within the one-year follow-up period, and those who underwent re-operation for any reason during the study period. A total of 84 patients underwent MEL during the study period. Patients meeting exclusion criteria were excluded from the correlation analyses involving those outcomes. Sample sizes vary by analysis due to outcome-specific missingness; the number of observations is reported in each table. The number of excluded patients is detailed in the Results section.

Surgical procedure

All procedures were MELs performed by board-certified spine surgeons using a standardized decompression technique. Multilevel surgery and concomitant procedures followed clinical indications.

Outcome measures

Preoperatively and at one year after surgery, we assessed lower back pain, buttock/leg pain, and leg numbness using 0-100 Visual Analogue Scales (VAS); disability using the RMDQ and the ODI; activities of daily living and bladder function using selected items from the JOA scale; and five domains of the JOABPEQ (lumbar function, pain-related impairment, walking ability, social life, and psychological impairment). For interpretability: higher VAS, RMDQ, and ODI indicate greater symptom burden or disability, whereas higher JOABPEQ scores indicate better status.

Patient satisfaction

Satisfaction at one year was captured by a single recommendation-level item asking whether the patient would recommend the procedure to others (five ordered response options). This item was analyzed on its original ordinal scale. The wording of the item and the five response options are shown in Table 1.

**Table 1:** Initial patient satisfaction survey: wording of the recommendation-level item and its five ordered response options.

Question	Option
Question 1	Recommendation level: Would you recommend the same surgical procedure to someone close to you? (4 points)
	4 points: Strongly recommend
	3 points: Recommend
	2 points: Neutral
	1 point: Do not recommend
	0 points: Strongly do not recommend
Question 2	Which aspects of the surgical procedure met your expectations? (Multiple answers allowed)
	1. Symptom improvement (e.g., pain, numbness, mobility, and other symptoms)
	2. Perioperative pain management (e.g., pain at the surgical site)
	3. Financial aspects related to the surgery and hospitalization
	4. Interaction with medical personnel during hospitalization (e.g., physicians, nurses, rehabilitation therapists, pharmacists, administrative staff)
	5. None
Question 3	Which aspects of the surgical procedure did not meet your expectations? (Multiple answers allowed)
	1. Symptom improvement (e.g., pain, numbness, mobility, and other symptoms)
	2. Perioperative pain management (e.g., pain at the surgical site)
	3. Financial aspects related to the surgery and hospitalization
	4. Interaction with medical personnel during hospitalization (e.g., physicians, nurses, rehabilitation therapists, pharmacists, administrative staff)
	5. None

Statistical analysis

Continuous variables are presented as mean ± standard deviation where appropriate. Pre-post comparisons used paired-sample t-tests when the distribution of paired differences approximated normality and Wilcoxon signed-rank tests otherwise; corresponding test statistics (t or W) are reported in the tables together with p-values. Between-group categorical comparisons, when applicable, used chi-square tests with the chi-square statistic reported. Associations between the one-year recommendation level and postoperative scores were estimated using Pearson correlation coefficients; correlation tables report r, p-values, and the number of complete observations. All tests were two-tailed with a significance threshold of 0.05. Given the exploratory nature of the analyses, no adjustment was made for multiple comparisons. Analyses were performed on a complete-case basis for each outcome. All analyses were performed using R version 4.4.1 (The R Core Team, R Foundation for Statistical Computing, Vienna, Austria).

Ethics

The study protocol was approved by the institutional review board of Kyushu Central Hospital, Fukuoka, Japan (approval number: 314). Owing to the retrospective design using de-identified data, the requirement for individual informed consent was waived in accordance with local regulations. The study adhered to the principles of the Declaration of Helsinki.

## Results

Patient characteristics

The patient demographic and clinical characteristics are listed in Table 2. A total of 84 patients underwent MEL during the study period. No patients were excluded. The final study cohort consisted of 84 patients (mean age: 70.7 ± 10.5 years; 49 males and 35 females). The number of vertebral levels involved in the surgery was one in 32 patients, two in 40 patients, and three in 12 patients. The average duration of surgery was 139.5 ± 53.8 minutes. There were no major intraoperative complications.

**Table 2:** Demographic and clinical characteristics of included patients JOABPEQ: Japanese Orthopaedic Association Back Pain Evaluation Questionnaire; VAS: Visual Analogue Scale; RMDQ: Roland-Morris Disability Questionnaire; JOA: Japanese Orthopaedic Association

Variable	Value (mean ± SD)
Age (years)	70.7 ± 10.5
Sex (male/female)	49/35
Height (cm)	160.4 ± 9.7
Weight (kg)	63.0 ± 15.0
BMI (kg/m²)	24.3 ± 4.5
Operated spinal levels	
Single level	32
- L1/2	1
- L2/3	1
- L3/4	4
- L4/5	24
- L5/S	2
Two levels	40
- L2/3/4	5
- L3/4/5	26
- L4/5/S	9
Three levels	12
- L2/3/4/5	8
- L3/4/5/S	4
Surgical duration (minutes)	139.5 ± 53.8
JOABPEQ: Lumbar function	43.4 ± 36.5
JOABPEQ: Pain-related impairment	54.3 ± 27.9
JOABPEQ: Walking ability	28.0 ± 23.2
JOABPEQ: Social life	38.3 ± 19.0
JOABPEQ: Psychological impairment	46.0 ± 13.6
VAS: Lower back pain (0–100)	53.9 ± 28.9
VAS: Leg pain (0–100)	64.0 ± 30.3
VAS: Leg numbness (0–100)	67.6 ± 26.8
RMDQ	12.4 ± 5.0
JOA: Activities of daily living	7.0 ± 3.0
JOA: Bladder function	-1.6 ± 1.7
Oswestry Disability Index	44.0 ± 15.1

Pre- to postoperative changes in lumbar spine scoring systems

The pre- to postoperative changes in lumbar spine scores are presented in Table 3. Significant improvements were observed across patient-reported outcomes and clinical scores. For example, the average VAS score for lower back pain decreased from 53.9 ± 28.9 preoperatively to 21.7 ± 24.1 postoperatively (Wilcoxon W = 155.00, p = 7.8 × 10⁻⁹), and the average RMDQ score improved from 12.4 ± 5.0 preoperatively to 5.7 ± 5.5 postoperatively (paired t = -8.51, p = 1.39 × 10⁻¹²). All five JOABPEQ domains (lumbar function, pain-related impairment, walking ability, social life, and psychological impairment) showed clear postoperative gains, and improvements were also evident in JOA activities of daily living and bladder function. Detailed statistics, including test statistics and p-values, are shown in Table 3.

**Table 3:** Pre- and postoperative scores with test statistics Values are mean ± SD. P-values from paired-sample t-tests (t) or Wilcoxon signed-rank tests (W), selected according to the distribution of paired differences. The “Test statistic” column reports t or Wilcoxon W as applicable. JOABPEQ: Japanese Orthopaedic Association Back Pain Evaluation Questionnaire; VAS: Visual Analogue Scale; RMDQ: Roland-Morris Disability Questionnaire; JOA: Japanese Orthopaedic Association

Scoring System/Measure	Preoperative (mean ± SD)	Postoperative (mean ± SD)	Test statistic	p-value
JOABPEQ: Lumbar function	43.4 ± 36.5	78.4 ± 29.1	W = 162.00	7.2×10⁻⁸
JOABPEQ: Pain-related impairment	54.3 ± 27.9	75.2 ± 25.5	W = 286.50	2.1×10⁻⁶
JOABPEQ: Walking ability	28.0 ± 23.2	67.4 ± 33.5	t = 9.74	7.7×10⁻¹⁵
JOABPEQ: Social life	38.3 ± 19.0	62.0 ± 26.2	t = 7.34	2.6×10⁻¹⁰
JOABPEQ: Psychological impairment	46.0 ± 13.6	55.3 ± 19.1	W = 587.50	1.26×10⁻⁴
VAS: Lower back pain (0–100)	53.9 ± 28.9	21.7 ± 24.1	W = 155.00	7.8×10⁻⁹
VAS: Leg pain (0–100)	64.0 ± 30.3	24.2 ± 24.3	W = 247.50	7.7×10⁻¹⁰
VAS: Leg numbness (0–100)	67.6 ± 26.8	21.5 ± 21.1	W = 77.50	1.30×10⁻¹¹
RMDQ	12.4 ± 5.0	5.7 ± 5.5	t = -8.51	1.39×10⁻¹²
JOA: Activities of daily living	7.0 ± 3.0	10.4 ± 3.1	W = 126.00	8.8×10⁻¹¹
JOA: Bladder function	-1.6 ± 1.7	-1.2 ± 1.6	W = 22.50	1.84×10⁻²
Oswestry Disability Index	44.0 ± 15.1	22.1 ± 17.9	t = -9.43	2.97×10⁻¹⁴

Patient satisfaction survey

The patient satisfaction survey detailed responses to three main questions: recommendation level, aspects of the surgery that met expectations, and aspects that did not meet expectations. The average score for the recommendation level (question 1) was 2.8 out of 4, indicating a high level of satisfaction. For question 2, which allowed multiple responses, 56 patients (66.7%) indicated improvement in symptoms as the most satisfying outcome. For question 3, 29 patients (34.5%) reported insufficient improvement in symptoms such as numbness and walking ability as their primary dissatisfaction. The detailed results are shown in Table 4. Table 5 categorizes the responses regarding postoperative symptom improvement.

**Table 4:** Correlation between one-year recommendation level and postoperative scores Pearson correlation coefficients (r) with two-tailed p-values. Positive r indicates a higher score associated with a higher recommendation level; negative r indicates an inverse association. Recommendation level corresponds to the satisfaction “recommendation” item. N indicates the number of patients with non-missing data for each correlation. JOABPEQ: Japanese Orthopaedic Association Back Pain Evaluation Questionnaire; VAS: Visual Analogue Scale; RMDQ: Roland-Morris Disability Questionnaire; JOA: Japanese Orthopaedic Association

Postoperative Variable	Pearson r	p-value	N
JOABPEQ: Lumbar function	0.21	0.073	74
JOABPEQ: Pain-related impairment	0.32	0.006	74
JOABPEQ: Walking ability	0.33	0.0038	74
JOABPEQ: Social life	0.37	0.0012	74
JOABPEQ: Psychological impairment	0.36	0.0017	74
VAS: Lower back pain (0–100)	-0.37	0.001	74
VAS: Leg pain (0–100)	0.24	0.027	82
VAS: Leg numbness (0–100)	-0.27	0.02	74
RMDQ	-0.33	0.004	76
JOA: Activities of daily living	0.2	0.078	75
JOA: Bladder function	0.34	0.0033	73
Oswestry Disability Index	-0.26	0.022	75

**Table 5:** Symptom improvement categories

Aspect of Surgery	Number of Responses
Pain improvement	49
Numbness improvement	28
Walking ability improvement	37
Perioperative pain	9
Financial aspects	5
Interaction with medical staff	25
None	11

Correlation between various scores at one year post surgery and recommendation levels

There were significant correlations between patient satisfaction (recommendation level) and several postoperative scores. Inverse associations were observed for residual lower back pain on VAS and disability on the RMDQ and ODI, whereas positive associations were observed for the JOABPEQ domains of social life, psychological impairment, walking ability, and pain-related impairment, and for bladder function. Some domains, including lumbar function and activities of daily living, showed weak or non-significant associations. Detailed coefficients, p-values, and sample sizes are summarized in Table 4.

## Discussion

The etiology of lower back pain associated with LSS includes discogenic and facet joint origins, abnormalities in spinal alignment, instability, nerve compression, and back muscle atrophy. Konno et al. reported that radicular lower back pain resolves with nerve root blocks, suggesting that pain is perceived due to nerve root or surrounding dura stimulation [12]. While it is known that pain relief after decompression surgery is influenced by factors such as sex and preoperative lower back pain severity, no clear predictors have been established [13].

Our study investigated the correlation between outcomes after MEL for LSS and patient-reported satisfaction at one year. We found that postoperative patient satisfaction (recommendation level) was inversely associated with residual lower back pain on VAS and with disability on the RMDQ and ODI and positively associated with the JOABPEQ domains of social and psychological impairment, walking ability, and pain-related impairment, as well as with bladder function. These findings indicate that satisfaction aligns most closely with the postoperative symptom and function profile rather than any single baseline factor, underscoring that the state patients achieve after surgery is pivotal for how they appraise success. This observation is compatible with prior work suggesting that baseline burden can shape perceived benefit [2], even as definitive preoperative predictors remain elusive [13].

Recent studies have highlighted the usefulness of the JOABPEQ in capturing patient-specific quality of life measures for lumbar diseases [4]. In our cohort, the JOABPEQ domains of social life and psychological impairment, together with walking ability and pain-related impairment, showed the strongest positive correlations with recommendation level, reinforcing the instrument’s capacity to reflect aspects of recovery that matter to patients in daily life [4].

At the same time, our results suggest that existing lumbar spine scoring systems, such as the VAS, RMDQ, ODI, and JOABPEQ, while valuable for documenting clinical improvement, may not fully evaluate patient satisfaction. The weak or non-significant associations observed for some domains (e.g., lumbar function and activities of daily living) illustrate that satisfaction is influenced by multiple determinants beyond symptom intensity and basic function alone [9]. Therefore, it is essential to implement separate, patient-centered satisfaction measures alongside standard instruments to obtain a more accurate understanding of patient outcomes and to address expectations and subjective experiences that traditional scales may miss [14].

Previous studies have indicated correlations between pain and treatment satisfaction and associations between psychosocial factors and patient satisfaction [4]. Our findings align with these observations, demonstrating significant correlations between postoperative satisfaction and scores related to pain relief as well as social and psychological functioning. Although numbness also correlated with satisfaction, its impact was comparatively smaller, which is consistent with prior work [4].

In the patient satisfaction survey, the recommendation level was generally favorable, and a substantial proportion of patients (66.7%) identified symptom improvement as the most satisfying outcome, whereas 34.5% reported insufficient improvement, particularly for numbness and walking ability, as their primary dissatisfaction. Patients also indicated dissatisfaction related to postoperative surgical site pain and interactions with medical staff during hospitalization. These responses suggest that satisfaction may be influenced by factors not fully quantified by existing scoring systems, implying that reliance on such systems alone may be insufficient to evaluate the primary goal of surgery-patient satisfaction [15]. Accordingly, comprehensive assessment frameworks that incorporate psychological and other patient-reported factors are warranted to capture the full patient experience after decompression surgery [16].

This study has several limitations that should be considered. It was a retrospective single-center analysis with a relatively small sample size (n=84), which may limit generalizability and statistical power. The satisfaction endpoint relied on a single recommendation-level item at one year and is susceptible to response and recall bias. Missing data reduced the number of complete cases for some correlations, and multiple comparisons raise the possibility of type I error. Pearson’s correlations assume linear relationships and cannot account for unmeasured confounding, including psychosocial factors not captured by the instruments. Finally, instrument heterogeneity and differing score directions may constrain direct comparability across measures.

## Conclusions

In this single-center cohort undergoing MEL for LSS, one-year patient satisfaction, assessed by a recommendation-level item, was moderately associated with postoperative pain relief and with social and psychological functioning and walking ability on standard instruments, and inversely associated with residual pain and disability. These findings indicate that conventional outcome scores capture important but incomplete dimensions of what patients value after surgery. Incorporating a brief, patient-reported satisfaction measure alongside established scores may provide a more balanced appraisal of recovery and support shared decision-making in routine practice. Prospective studies are warranted to confirm these associations and to refine how satisfaction is integrated into outcome evaluation.
